# Mass Load Distribution Dependence of Mass Sensitivity of Magnetoelastic Sensors under Different Resonance Modes

**DOI:** 10.3390/s150820267

**Published:** 2015-08-18

**Authors:** Kewei Zhang, Lin Zhang, Yuesheng Chai

**Affiliations:** 1 School of Materials Science and Engineering, Taiyuan University of Science and Technology, Taiyuan 030024, China; E-Mail: cys5912@126.com; 2 Materials Research and Education Center, Auburn University, Auburn, AL 36849, USA; E-Mail: lzz0002@auburn.edu

**Keywords:** magnetoelastic sensor, mass sensitivity, resonance mode, mass load distribution

## Abstract

Magnetoelastic sensors as an important type of acoustic wave sensors have shown great promise for a variety of applications. Mass sensitivity is a key parameter to characterize its performance. In this work, the effects of mass load distribution on the mass sensitivity of a magnetoelastic sensor under different resonance modes were theoretically investigated using the modal analysis method. The results show that the mass sensitivity and “nodal point” positions are related to the point displacement, which is determined by the motion patterns. The motion patterns are affected by resonance modes and mass load distribution. Asymmetrical mass load distribution causes the motion patterns lose symmetry and leads to the shift of “nodal point”. The mass sensitivity changing with mass load distribution behaves like a sine wave with decaying amplitude and the minimum mass sensitivity appears at the first valley. This study provides certain theoretical guidance for optimizing the mass sensitivity of a magnetoelastic sensor or other acoustic wave based sensors.

## 1. Introduction

In the past decades, magnetoelastic sensors (MS) have shown great potential in a wide range of applications due to the advantages of wireless in detection, easiness in operation, and low cost [1,2]. A typical MS is made of strip-shaped soft ferromagnetic ribbons or wires which can be excited to vibrate by applied time-varying magnetic field due to the magnetostriction effect [1]. In other words, an MS can act as a resonator whose resonance frequency is usually used as the detection signal for sensor application [1]. As an acoustic wave device, an MS is sensitive to the mass attached on its surface, which leads to the change in its resonance frequency [2,3]. Therefore, a variety of MS for the detection of pathogens, toxins and other chemicals have been developed based on the biochemical/physical reaction induced mass detection [2,3,4,5,6,7,8,9,10,11,12,13,14,15]. For a mass detection based acoustic wave sensor, a critical parameter to characterize its performance is mass sensitivity (*S_m_*), which is defined as the change in the resonance frequency per unit mass load [16]. The mass sensitivity can be well determined if the mass is uniformly attached on the whole MS surface [17]. For instance, the Metglas^TM^ 2826MB (a commercial soft magnetic alloy) based MS in the length of 1 mm for the first order resonance has the mass sensitivity of ~50 Hz/ng [1,2,3].

In recent years, some researchers have found that mass sensitivity is strongly dependent on the mass load position/distribution and the resonance modes of an MS. Ramasy *et al.* reported that the same mass load attached at different positions on an MS can give rise to different mass sensitivity [18]. Particularly, there exists a “nodal point” where the point displacement as well as the mass sensitivity is zero when the mass is loaded on it. Li *et al.* investigated the effect of resonance modes on the mass sensitivity for the manner of symmetrical mass distribution and the experimental results showed that the mass sensitivity is dependent on the oscillation amplitude where the mass is attached [16]. The highest mass sensitivity is obtained when the mass is loaded at the points with the maximum oscillation amplitude, while the lowest sensitivity is obtained when the mass is loaded at the “nodal point” [16]. Particularly, the number and positions of the so called “blind points” (*i.e.*, “nodal points”) change with the resonance modes. It is worth noting that in most MS related studies, for example, magnetoelastic biosensors, the targets (e.g., bacteria) are designed to be immobilized over the whole surface of an MS [2,3,4,5,6,7,8,9,10,11,12]. This means that the mass sensitivity can be further improved if the manner of immobilization is well controlled.

Though some experiments and simulations have been carried out to study the behavior of mass sensitivity, few theoretical studies were done on the fundamentals behind the experimental phenomena. In our previous work, we theoretically derived the mass sensitivity and the “nodal point” position for different mass load distribution using a modal analysis method [19]. It is found that the mass sensitivity as well as the “nodal point” position changes with mass load distribution. Actually, in addition to MS, other types of acoustic wave sensors such as magnetoelastic micro-cantilevers (MSMCs) [20,21], piezoelectric [22,23,24] and mechanical cantilevers [25] also work in a similar principle with MS and thus they share the same issue on the mass sensitivity as well as the “nodal point”.

In this work, we focus on the MS and theoretically studied both the effect of mass load distribution and resonance modes on mass sensitivity of the MS. Based on the analysis on MS, it is anticipated to have certain theoretical guidance for the optimization of mass sensitivity for all acoustic wave based sensors.

## 2. Theory

**Figure 1:**
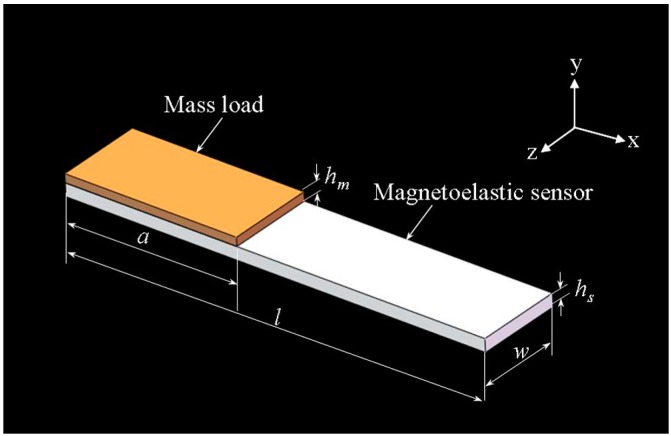
Illustration of a magnetoelastic sensor (MS) loaded with a layer of uniform mass where *l*, *w*, and *h_s_* represent the length, width and thickness of the MS; *a* (0 ≤ *a* ≤ *l*) and *h*_m_ represent the length and thickness of the mass load layer.

The MS is assumed as a free-standing strip-shaped elastic plate which is attached by a layer of uniform mass load as shown in Figure 1.

The kinetic energy (*T*) and potential energy (*V*) of the MS with the layer of mass load can be expressed as [19]:

(1) $$T=\frac{1}{2}\int_{0}^{l}\rho_{s}A_{s}{\left(\frac{\partial u\left(x,t\right)}{\partial t}\right)}^{2}dx+\frac{1}{2}\int_{0}^{a}\rho_{m}A_{m}{\left(\frac{\partial u\left(x,t\right)}{\partial t}\right)}^{2}dx$$

(2) $$V=\frac{1}{2}\int_{0}^{l}\frac{E_{s}}{1-\nu_{s}}A_{s}{\left(\frac{\partial u\left(x,t\right)}{\partial x}\right)}^{2}dx+\int_{0}^{a}\frac{E_{m}}{1-\nu_{m}}A_{m}{\left(\frac{\partial u\left(x,t\right)}{\partial x}\right)}^{2}dx$$

where *ρ_s_*, *E_s_*, *ν_s_*, and *A_s_* represent the density, Young’s modulus, Poisson’s ratio and cross-sectional area (*w* × *h_s_*) of the MS, respectively; *ρ_m_*, *E_s_*, *ν_s_* and *A_m_* represent the density, Young’s modulus, Poisson’s ratio and cross-sectional area (*w* × *h_m_*) of the mass load layer. In general, the second term in Equation (2) can be neglected [19].

The displacement function for the *n*th-order resonance is expressed as [19]:

(3) $$u_{n}\left(x,t\right)=\phi_{1}\left(x\right)q_{1n}\left(t\right)+\phi_{2}\left(x\right)q_{2n}\left(t\right)+...+\phi_{n}\left(x\right)q_{nn}\left(t\right)$$

where *φ_n_*(*x*) is the mode shape function of the *n*th-order resonance of the MS; *q_nn_*(*t*) is the element of generalized coordinate which is a *n* × *n* matrix. 

The *n*th-order mode shape function for a free-standing strip-shaped MS are known as [26]

*φ_n_* = cos(*n*π/*l*) (*n* = 1, 2, 3,…) (14)

In this study, the first four mode shape functions are used and studied. The governing vibration equation is derived based on the Lagrange equation as expressed as [19]

(5) $$\left(K-{\omega_{n}}^{2}M\right)q=0$$

where ***K***, ***M*** and *ω_n_* represent stiffness matrix, mass matrix and generalized eigenvalue. The element *M_ij_* in ***M*** is defined as $M_{ij}=\int_{0}^{a}\rho_{m}A_{m}\phi_{i}\left(x\right)\phi_{j}\left(x\right)dx$ and the element *K_ij_* in ***K*** is defined as $K_{ij}=\int_{0}^{l}\frac{E}{1-\nu }A_{s}\phi_{i}^{'}\left(x\right)\phi_{j}^{'}\left(x\right)dx$.

By solving Equation (5), *ω_n_* can be obtained and thus the *n*th-order resonance frequency can be calculated by

*f_n_* = *ω_n_*/2π (*n* = 1, 2, 3,…) (6)

Hence, the change in the *n*th-order resonance frequency (Δ*f_n_*) is obtained by

Δ*f_n_* = *f_ns_* − *f_nm_* (*n* = 1, 2, 3,…) (7)

where *f*_ns_ and *f*_nm_ represent the *n*th-order resonance frequency for the MS without and with mass load (*i.e.*, *a*/*l*). 

The parameters for the MS and the mass load used in this study are listed in Table 1. Metglas^TM^ 2826 MB (widely used for sensor application) is selected as the MS material. Twenty different mass load conditions (*i.e.*, *a*/*l*) are used. All the calculations are carried out by MATLAB software.

**Table 1:** The parameters for the MS and the mass load used in this study [27].

	Symbol	Unit	Value
Young’s modulus	*E*	GPa	105
Density	*ρ_s_*	kg/m^3^	7.9 × 10^3^
Poisson’s ratio	*ν*	-	0.33
Length	*l*	mm	1
Width	*w*	mm	0.2
Thickness	*h_s_*	μm	15
*a/l*	-	-	0.1, …, 1.0
Mass ratio per unit length	*ρ_m_A_m_*/*ρ_s_A_s_*	-	10^−3^

## 3. Results and Discussion

### 3.1. The Effect on Motion Patterns and “Nodal Points”

**Figure 2:**
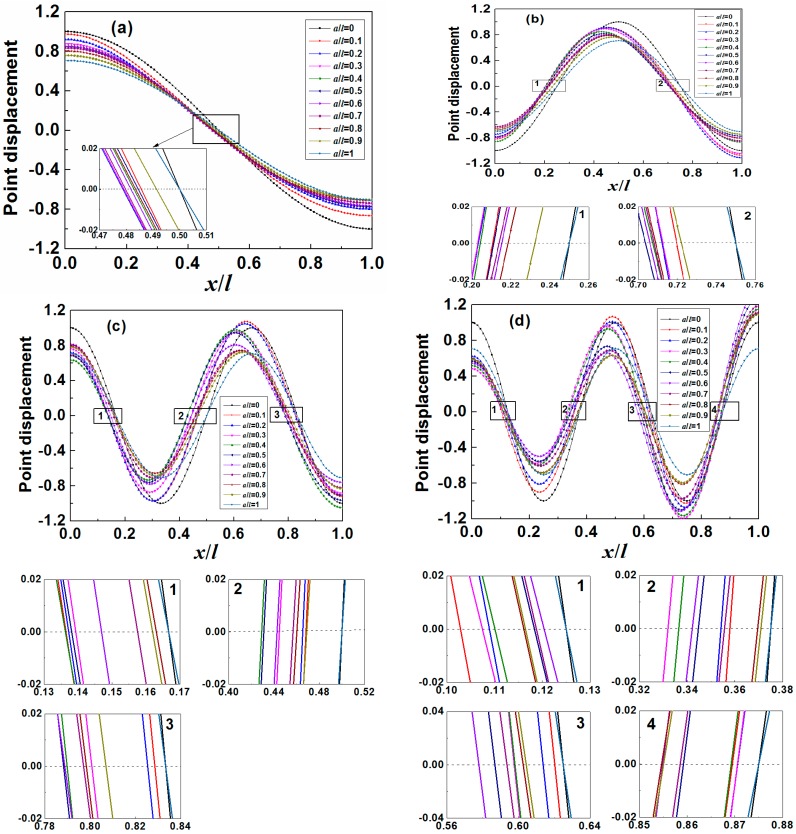
The motion patterns of an MS with different mass load distribution (*a*/*l*) for (**a**) first order resonance mode; (**b**) second order resonance mode; (**c**) third order resonance mode; and (**d**) fourth order resonance mode. Here, *ρ_m_A_m_*/*ρ_s_A_s_* = 1 is used for easy distinction of each pattern.

From Equation (3) the motion pattern of the MS with any given mass load distribution (*i.e.*, *a*/*l*) and resonance mode can be determined as shown in Figure 2 where the *x*-coordinate (*i.e.*, *x*/*l*) at zero point displacement corresponds to the “nodal point”. Clearly, it is found that the motion patterns as well as the “nodal point” are affected by the mass load distribution and resonance modes. Particularly, for the mass load conditions of *a*/*l* = 0 and *a*/*l* = 1, the motion patterns exhibit the features of center-symmetry for odd-order resonance modes and mirror-symmetry for even-order resonance modes. Moreover, from the insert figures, it can be obtained that the “nodal point” positions for the above two mass load conditions are the same and the corresponding “nodal points” for the first four order resonance modes are listed in Table 2. On the other hand, asymmetrical mass load distribution (*i.e.*, 0 < *a*/*l* < 1) causes motion patterns lose symmetry that is one reason that the “nodal point” shifts with the changing *a*/*l*.

**Table 2:** The “nodal point” position for the MS with *a*/*l* = 0 and *a*/*l* = 1.

Resonance Order	Nodal Point Position(s) at *x* =
1	*l*/2
2	*l*/4, 3*l*/4
3	*l*/6, *l*/2, 5*l*/6
4	*l*/8, 3*l*/8, 5*l*/8, 7*l*/8

**Figure 3:**
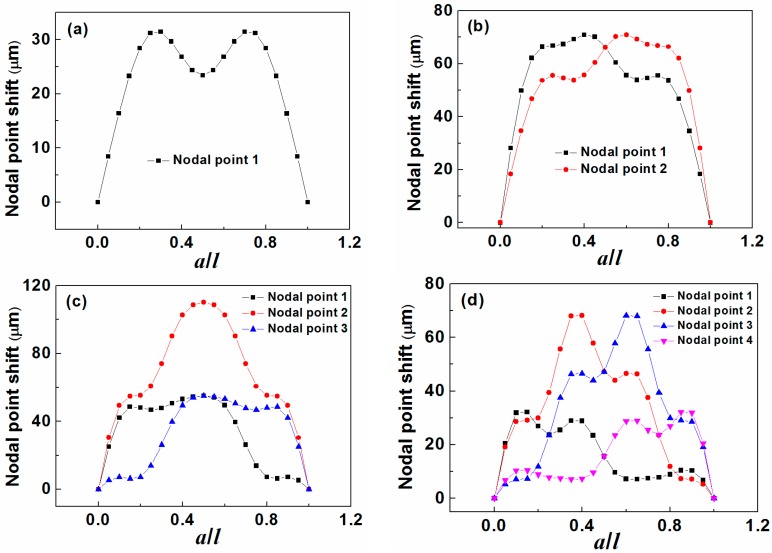
The plot of “nodal point” shift changing with mass load distribution (*a*/*l*) under (**a**) first order resonance mode; (**b**) second order resonance mode; (**c**) third order resonance mode; and (**d**) fourth order resonance mode.

From the motion patterns, we can easily determine the “nodal point” shift which is the difference between the “nodal point” position (*x*/*l*) without mass load (*a*/*l* = 0) and with mass load (*a*/*l* ≠ 0). The plots of “nodal point” shift as the function of *a*/*l* for different resonance modes are shown in Figure 3, where significant difference is observed between each curve. For example, for the third order resonance mode, the curve for “nodal point 2” is self-symmetrical about *a*/*l* = 0.5 while the curves for “nodal point 1” and “nodal point 3” are mirror-symmetrical about *a*/*l* = 0.5. From Figure 3a–d, it is further concluded that for any one odd-order resonance mode (2*n* − 1, *n* = 1, 2, 3, … ), there is always one curve((*n*)th “nodal point”) self-symmetrical about *a*/*l* = 0.5 and (*n* − 1) pair of curves((*q*)th “nodal point” and (2*n* − *q*)th “nodal point” where *q* = 1, 2, 3, …, *n* − 1) mirror-symmetrical about *a*/*l* = 0.5 while for even-order (2*m*, *m* = 1, 2, 3, … ) resonance mode, there are only *m* pair of curves((*p +* 1)th “nodal point” and (2*m* − *p*)th “nodal point” where *p* = 0, 1, 2, …, *m* − 1) mirror-symmetrical about *a*/*l* = 0.5.

### 3.2. The Effect on Frequency Shift

**Figure 4:**
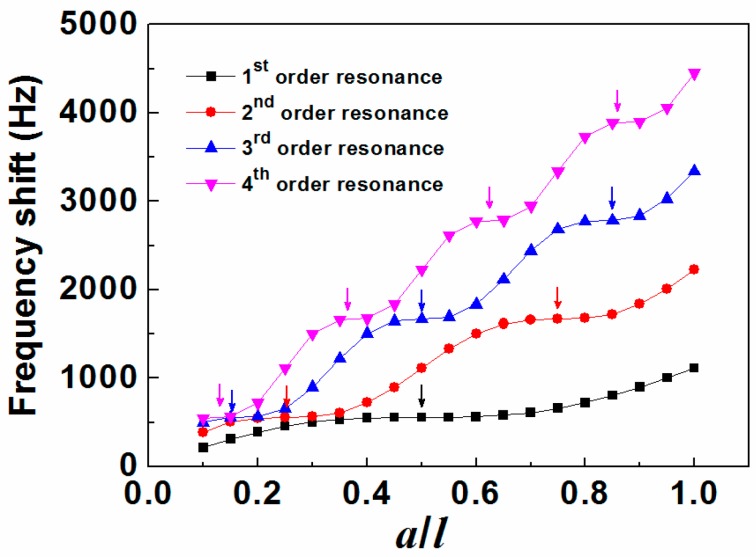
Mass load distribution (*a*/*l*) dependence of frequency shift of the magnetoelastic sensor under different order resonance modes.

The frequency shift changed with mass load distribution under different order resonance modes are shown in Figure 4. It indicates that the frequency shift increases monotonously but nonlinearly as *a*/*l* increasing for all the resonance modes. Moreover, all the curves behave like stairs but with different number of “platforms” as marked by the arrows in different color. Actually, the so called “platforms” are not exactly parallel to *x*-axis but with very slight slope. Here we define the “platform” as the span where the slope of frequency shift versus *a*/*l* is the local minimum. Clearly, the span of the platforms decreases as the increasing resonance order. The number of “platforms” for each resonance mode is also found the same as that of the resonance order as well as the number of “nodal points”. In addition, each “nodal point” is found near the middle of a “platform”. For example, for the second order resonance mode, the two “nodal points” are 1/4 length away from the two ends of the MS (*i.e.*, ~1/4*l* and ~3/4*l*), respectively while the two platforms are found in the range of 0.2*l* to 0.3*l* with only a few frequency shift changing from ~549 Hz to ~564 Hz and 0.7*l* to 0.8*l* with the frequency shift changing from ~1662 Hz to ~1676 Hz. This indicates that mass loaded near the “nodal points” has little effect on the frequency shift or little contribution to the mass sensitivity. This is consistent with the previous experimental results [16].

### 3.3. The Effect on Mass Sensitivity

For an MS with the mass load condition of *a*/*l* = 1, the mass sensitivity can be calculated by the well-known equation [17]

(8) $$S_{m}\approx \frac{f_{n}}{2m_{s}}\;(\Delta m<<m_{s})$$

where *m*_s_ is the mass of the MS and *f_n_* is the *n*th-order resonance frequency of the MS without mass load which is expressed as [28]

(9) $$f_{n}=\frac{n}{2l}\sqrt{\frac{E}{\rho (1-\nu )}}\;(n=\text{1, 2, 3}...)$$

where *l*, *E*, *ρ*, and *ν* represent the length, Young’s modulus, density and Poisson’s ratio of the MS, respectively.

Since Δ*m*/*m_s_* is 10^−3^ (*i.e.*, Δ*m* << *m_s_*) in this study, the mass sensitivity obtained from Equation (8) is reasonable. As a comparison, the mass sensitivity is also calculated using the proposed method in this study and the results are listed in Table 2. Obviously, the same results indicate the validity of the methodology used in this study.

**Table 3:** Comparison of the mass sensitivity for *a*/*l* =1 calculated by the two methods.

Resonance Order	Mass Sensitivity (Hz/ng)	
	Methodology Used in This Study	Equations (8) and (9)
1	47	47
2	94	94
3	141	141
4	188	188

**Figure 5:**
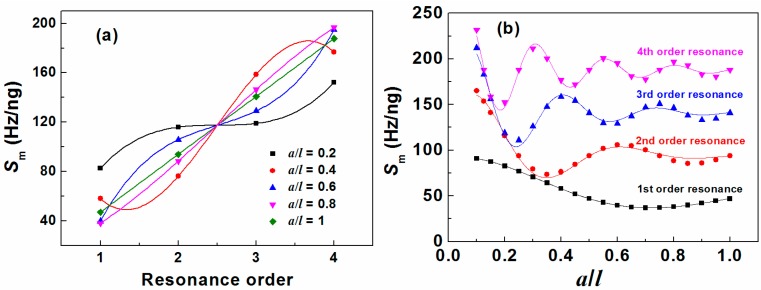
Mass sensitivity (*S*_m_) as the function of (**a**) resonance order; (**b**) mass distribution.

It is seen from Table 3 that the mass sensitivity linearly increases with resonance order in the mass load condition of *a*/*l* = 1 but nonlinearly increases for the condition 0 < *a*/*l* < 1 as shown in Figure 5a. Obviously, the higher order resonance is preferred to obtain a higher mass sensitivity. However, a higher resonance order corresponds to a higher applied frequency, which would lead to the increasing eddy current damping. As a result, the resonance signal is weakened even to none as the resonance frequency increasing. Therefore, there is a balance between the mass sensitivity and the signal strength. In addition, it is interesting to find that all the fitting curves cross at the theoretically non-exsistent resonance order of 2.5 in Figure 5a. On the other hand, the mass sensitivity changing with *a*/*l* behave like sine waves with decaying amplitude and the number of valleys for each resonance mode is the same as the corresponding resonance order as well as the number of “nodal points” as shown in Figure 5b. Interestingly, it is found that the *S_m_* versus *a*/*l* curves can be fitted well with

(10) $$S_{m}=S_{m0}+Ae^{-\frac{x}{B}}\sin(\pi \frac{x-C}{D})$$

where *S_m_*_0_, *A*, *B*, *C* and *D* are constant; *S_m_* represents the mass sensitivity and *x* represents *a*/*l*.

**Figure 6:**
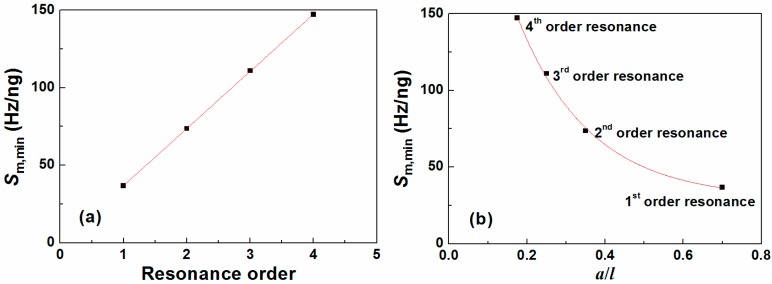
(**a**) The minimum mass sensitivity *versus* the resonance order where the data is fitted by a linear function; (**b**) The minimum mass sensitivity *versus* mass load distribution (*a*/*l*) where the data is fitted by an exponent decaying function.

It is also found that the minimum mass sensitivity (*S*_m,min_) for each resonance mode appears at the first valley but the corresponding mass loading conditions *a*/*l* are different. Figure 6a shows the *S*_m,min_ as the function of the resonance orders from which a linear relationship is observed. Based on the linear fitting function, the minimum mass sensitivity for any order resonance can be determined. To further obtain the corresponding value of *a*/*l*, the *S*_m,min_ as the function of *a*/*l* for different order resonances is plotted as shown in Figure 6b. Clearly, the value of *a*/*l* for the *S*_m,min_ seems to decrease with increasing resonance order. In addition, it is also found that the data can be well fitted by

(11) $$S_{m,\min}=y_{0}+Ae^{\left(-x/t\right)}$$

where *y*_0_, *A*, *t* are constant; *x* represents *a*/*l*.

By combining Equation (9) and the linear fitting function for the data in Figure 6a, the value of *a*/*l* for the minimum mass sensitivity for any order resonance mode can be determined.

## 4. Conclusions

A modal analysis method is applied to study the effect of mass load distribution and resonance modes on mass sensitivity of a magnetoelastic sensor. Based on the results, several points are concluded:

1. The motion pattern of a magnetoelastic sensor is dependent on its resonance mode and the mass load distribution. The mass sensitivity and “nodal point” position are related to the point displacement, which is determined by the motion pattern. Asymmetrical mass load distribution causes the motion patterns lose symmetry and leads to the shift in “nodal point”.
2. For any one odd-order resonance mode (2*n* − 1, *n* = 1, 2, 3, … ), there is always one curve((*n*)th “nodal point”) self-symmetrical about *a*/*l* = 0.5 and (*n* − 1) pair of curves((*q*)th “nodal point” and (2*n* − *q*)th “nodal point” where *q* = 1, 2, 3, …, *n* − 1) mirror-symmetrical about *a*/*l* = 0.5 while for even-order (2*m*, *m* = 1, 2, 3, … ) resonance mode, there are only *m* pair of curves((*p +* 1)th “nodal point” and (2*m* − *p*)th “nodal point” where *p* = 0, 1, 2, …, *m* − 1) mirror-symmetrical about *a*/*l* = 0.5.
3. Mass loaded near the “nodal point” has little contribution on the resonance frequency shift as well as the mass sensitivity.
4. Mass sensitivity linearly increases with resonance order for symmetrical mass load distribution but nonlinearly varies for asymmetrical mass load distribution, which is attributed to the loss of symmetry of motion patterns.
5. The mass sensitivity as the function of *a*/*l* behaves like sine waves with decaying amplitude. The minimum mass sensitivity appears at the first valley and is linearly proportional to the resonance order.

The results are anticipated to have certain theoretical guidance for the optimization design of mass sensitivity for other acoustic wave based sensors.
